# Rapid detection of *Pseudomonas aeruginosa* by glycerol one-pot RAA/CRISPR-Cas12a method

**DOI:** 10.3389/fchem.2025.1654270

**Published:** 2025-07-25

**Authors:** Lijian Wei, Shihua Luo, Weijie Zhou, Baoyan Ren, Miao Li, Lina Liang, Xuebin Li, Guijiang Wei

**Affiliations:** ^1^Center for Medical Laboratory Science, Affiliated Hospital of Youjiang Medical University for Nationalities, Guangxi, China; ^2^ Key Laboratory of Research on Clinical Molecular Diagnosis for High Incidence Diseases in Western Guangxi of Guangxi Higher Education Institutions, Guangxi, China; ^3^ Baise Key Laboratory for Precise Genetic Testing of Long-dwelling Nationalities, Guangxi, China; ^4^ Engineering Research Center of Guangxi Higher Education Institutions for Precise Genetic Testing of Long-dwelling Nationalities, Guangxi, China; ^5^ Guangxi Engineering Research Center for Precise Genetic Testing of Long-dwelling Nationalities, Guangxi, China; ^6^Department of Clinical Laboratory, Baise People’s Hospital, Affiliated Southwest Hospital of Youjiang Medical University for Nationalities, Guangxi, China; ^7^ Yaneng BlOscience (Shenzhen) Corporation, Guangdong, China; ^8^Modern Industrial College of Biomedicine and Great Health, Youjiang Medical University for Nationalities, Guangxi, China; ^9^Clinical Genome Center, Guangxi KingMed Diagnostics, Guangxi, China

**Keywords:** recombinase-aided amplification, glycerol one-pot CRISPR/Cas12a method, lateral flow chromatography strip, rapid detection, *Pseudomonas aeruginosa*

## Abstract

*Pseudomonas aeruginosa* (PA), an opportunistic pathogen commonly responsible for hospital-acquired infections, poses significant threats to human health. To enable rapid and reliable PA detection while effectively mitigating aerosol contamination risks inherent in conventional methods. We developed a glycerol one-pot Recombinase-aided Amplification (RAA)/CRISPR-Cas12a method. Four result reading methods were established: Fluorescence Detection (FD), Blue Light Irradiation Detection (BLD), and Ultraviolet Irradiation Detection (UID), as well as Lateral Flow Chromatography Strip (LFS). The glycerol one-pot RAA-CRISPR/Cas12a method demonstrated high specificity and sensitivity in detecting the PA-specific lasB gene. The detection limit reached 1.20 × 10^-4^ ng/μL (fluorescence-based) and 1.20 × 10^−3^ ng/μL (LFS-based). In validation against 64 clinical isolates, compared to conventional PCR, the assay achieved 100% sensitivity, specificity, and accuracy in lasB detection. In conclusion, the glycerol one-pot RAA/CRISPR-Cas12a method provides a rapid, sensitive, and straightforward platform, providing a promising approach for clinical diagnosis of PA and environmental surveillance applications.

## 1 Introduction

*Pseudomonas aeruginosa* (PA), a ubiquitous Gram-negative *bacillus*, exhibits exceptional environmental resilience characterized by high colonization potential on medical devices, human skin, and disinfectants. Its remarkable adaptability stems from rapid mutation rates, intrinsic multidrug resistance, and exceptional survival capabilities (Yu et al., 2025). Functioning as an opportunistic pathogen, PA causes significant hospital-acquired infections, particularly in immunocompromised individuals, posing severe threats to patient safety. Clinical manifestations include respiratory infections, bacteremia, urinary tract infections, burn wound sepsis, otitis media, and meningitis. etc (Xu et al., 2024). Current diagnostic approaches primarily encompass bacterial culture (the reference standard), quantitative real-time polymerase chain reaction (qRT-PCR), and mass spectrometry (Zhu et al., 2024). Despite its status as the gold standard, the conventional method demonstrates critical limitations. Culture-based identification necessitates 48–72 h for confirmation, often delaying critical therapeutic interventions (Zhang et al., 2024a). qRT-PCR and mass spectrometry methods demand sophisticated instrumentation and specialized personnel. Consequently, culture-dependent diagnosis frequently impedes timely antibiotic stewardship, adversely impacting patient outcomes (Liu et al., 2023). Although qRT-PCR offers high accuracy, it remains technically demanding, time-consuming, and infrastructure-intensive. This diagnostic landscape underscores the urgent need for simplified, rapid, sensitive, and specific detection platforms. Developing such assays is critical for achieving timely diagnosis of PA infections and implementing effective containment strategies to mitigate nosocomial transmission.

In recent years, to overcome the limitations of PCR, a variety of isothermal amplification methods have been created. Such as its strict thermal cycling requirements and long detection time. These methods include rolling circle amplification (RCA) (Long et al., 2025a), loop-mediated isothermal amplification (LAMP) (Zheng et al., 2023; Wang et al., 2025), recombinase polymerase amplification (RPA) (Yang et al., 2024a; Long et al., 2025b), and recombinase-aided amplification (RAA) (Kong et al., 2025; Liu et al., 2025). These emergent molecular diagnostic platforms have garnered significant interest as point-of-care tools due to their operational simplicity, cost-effectiveness, and high analytical sensitivity (Zheng et al., 2024), positioning them as viable alternatives with the potential to supersede conventional PCR in specific applications. CRISPR-Cas12a-coupled recombinase polymerase amplification (RPA) technology has been widely implemented for rapid pathogen detection (Munawar, 2022). This integrated system demonstrates outstanding diagnostic functions. The characteristics are: high specificity, high sensitivity, short reaction time, and high cost efficiency (Lesinski et al., 2024). RPA exhibits notable operational simplicity: Its isothermal amplification protocol eliminates thermocycling requirements, proceeding efficiently at constant temperatures (typically 37°C–42 °C) (). The system enables target detection within 30 min without sophisticated instrumentation. The CRISPR-Cas system comprises clustered regularly interspaced short palindromic repeats (CRISPR) and CRISPR-associated (Cas) nucleases (Fan et al., 2025). Its collateral cleavage activity provides powerful signal amplification, establishing it as a transformative biotechnology platform (Shi et al., 2021). Xu et al. (2023) demonstrated a LAMP/RPA-CRISPR-Cas12a-based lateral flow assay achieving *S. aureus* detection with sensitivities of 57.8 fg/μL and 6.7 × 10^2^ CFU/mL within 40 min. Zhou et al. (2024) developed a CRISPR-Cas12a-coupled RPA assay for rapid identification of crab pathogen genomic markers under isothermal conditions (37°C–40 °C), completing detection within 90 min with a sensitivity of 1.3 × 10^−6^ ng/μL.

Conventional two-step RPA/CRISPR-Cas12a assays necessitate sequential amplification and detection phases. This manual transfer step generates aerosols, elevates contamination risks, and increases false-positive potential due to nucleic acid cross-contamination. Integrating both reactions within a single closed vessel eliminates open-tube manipulations, substantially reducing contamination hazards and enhancing result reliability. To address the limitations of aerosol contamination and time-intensive workflows inherent in two-step approaches, integrated one-pot RPA/CRISPR-Cas12a platforms have emerged (Chen et al., 2018; Li et al., 2021). Tan et al. (2024a) developed a single-tube assay partitioning RPA and CRISPR components with paraffin wax. This platform demonstrated excellent analytical performance for simultaneously detecting 12 respiratory pathogens. Li et al. (2024) engineered a compartmentalized system with RPA reagents submerged under mineral oil at the tube base and CRISPR-Cas12a components attached to the inner cap. This design prevents pre-mixing while enabling subsequent reaction integration, minimizing aerosols, and reducing processing time. Hu et al. (2022) implemented a photo-controlled crRNA silencing strategy using photo-cleavable ligands (PC). Upon RPA completion, UV illumination releases functional crRNA, triggering CRISPR-Cas12a activation. This temporal separation eliminates cross-talk while optimizing reaction kinetics and reducing contamination. Hu et al. (2023) established a light-inducible detection platform employing caged crRNA, enabling rapid (<30 min) and accurate nucleic acid identification post-photolysis activation.

This study engineered glycerol one-pot RAA-CRISPR/Cas12a assay targeting the *P. aeruginosa-*specific lasB gene. The detection results were output as fluorescence signals and lateral flow chromatography strips. Integration of isothermal amplification with CRISPR-Cas12a detection within a sealed system simplifies the operation steps, and substantially reduces aerosol risk, rapid (<40 min) and ultrasensitive identification of *P. aeruginosa*. This approach facilitates expeditious pathogen identification, providing critical time advantages for initiating targeted antimicrobial therapy.

## 2 Materials and methods

### 2.1 Reagents and materials

Standard strains of *Pseudomonas aeruginosa* (ATCC 27853), *Klebsiella pneumoniae* (ATCC 700603), *Staphylococcus aureus* (ATCC 29213), *Escherichia coli* (ATCC 35218), *Enterococcus faecalis* (ATCC 29212), *Staphylococcus aureus* (ATCCBAA 1026), *Escherichia coli* (ATCC 25922), *Haemophilus haemolyticus* (ATCC 33390), *Streptococcus pneumoniae* (ATCC 49619); *Moraxella catarrhalis Stenotrophomonas maltophilia*, *Enterobacter cloacae*, *Acinetobacter baumannii*, *and Serratia marcescens* (isolated from clinical strains) were provided by the Department of Laboratory Medicine of the Affiliated Hospital of Youjiang Medical University for Nationalities and the Department of Laboratory Medicine of Baise People’s Hospital. *Enterococcus faecaium* was purchased from the Guangdong Microbial Culture Collection Center. The source information of the strains is shown in Supplementary Table S1. For testing of clinical samples, 45 clinical isolates of *P. aeruginosa* and 19 clinical isolates of other strains were collected from the Microbiology Unit of the Department of Laboratory Medicine and used for the evaluation of the effectiveness of clinical testing. All clinical strains were identified using the VITEK^®^2 Compact Microbial Identification System (Lyon, France).

The recombinase-aided amplification (RAA) Nucleic Acid Amplification Basic Kit was purchased from Hangzhou Heavy Measurement Biotechnology Co. Ltd (Hangzhou, China). EnGen®Lba Cas12a (Cpf1) and NEBuffer r2.1 were purchased from New England Biolabs. The 2 × Es Taq MasterMix (dye) for polymerase chain reaction (PCR) was purchased from Huabo Biotech (Jiangsu, China). Fluorescence readings were recorded using a Tenryu GenTiger 96E/96R qPCR analysis system (Xi’an, China). PAGE-purified oligonucleotides, RAA and PCR primers, and crRNAs were purchased from Jenis Biotech (Nanning, China), and single-stranded DNAs were purchased from Sangon Bioengineering Co. Tiosbio^®^ Cas12/13 test strips were purchased from Beijing Baoying Tonghui Biotechnology Co. Bacterial genomic DNA extraction kit was purchased from Tiangen Biochemical Technology (Beijing) Co.

### 2.2 Extraction of bacterial genomic DNA

Bacterial DNA was isolated following the protocol provided by the Bacterial DNA Extraction Kit (Tiangen, China). The concentration of the extracted DNA was assessed via a spectrophotometer. For the DNA samples to be considered suitable for subsequent analyses, their OD 260/280 ratios were required to fall within the range of 1.7–1.9. Any excess DNA was preserved at −20 °C for future use.

### 2.3 Basic principles of RAA nucleic acid amplification

Recombinase-aided amplification (RAA) is an isothermal nucleic acid amplification technique that exponentially amplifies target DNA sequences at constant temperatures (typically 37°C–42°C) using recombinase-primer complexes and accessory proteins (Zhao et al., 2025). The basic principles are as follows: 1) Formation of the complex: First, the recombinase binds to the primer, forming a primer-recombinase complex. The complex can recognize homologous sequences within the target DNA (Zhang et al., 2025a). 2) Opening of double-stranded DNA: When the complex finds the complementary sequences in the target DNA, the recombinase facilitates the separation of the double-stranded DNA, the complex is disassembled, and the primer and the target DNA base sequences are complementarily paired. 3) Strand stabilization and extension: The single-stranded binding protein (SSB) attaches to the open single-stranded DNA, maintaining the single-stranded stability. Subsequently, a Conjugate replacement polymerase connects to the 3 ends of the primer and synthesizes a new complementary strand based on the principle of base pairing. 4) Exponential amplification: Newly synthesized strands become templates for subsequent cycles, driving logarithmic target amplification through iterative repetition of this process (Xu et al., 2025).

### 2.4 Principles of CRISPR/Cas12a enzymatic cleavage

CRISPR-Cas12a is a programmable genome-editing tool that exhibits nonspecific collateral cleavage activity, which can be harnessed for signal amplification (Zheng et al., 2025). Cas12a is an RNA-mediated nucleic acid endonuclease. When crRNA identifies the aim sequence, it directs Cas12a to cleave DNA at the target site simultaneously. It also activates the non-specific nucleic acid endonuclease activity of Cas12a, which generates an arbitrary cutting activity of single-stranded DNA (Tan et al., 2024b). Target sequence recognition triggers Cas12a collateral cleavage function, which indiscriminately degrades nearby fluorescent reporter oligonucleotides, liberating fluorophores and generating detectable signals. The fluorescence signals are collected to ascertain the presence of the target DNA.

### 2.5 Design of RAA primers and crRNAs

Primers were designed following Recombinase-aided Amplification (RAA) specifications: length 28–35 nt, amplicon size 200–300 bp, and GC content 20%–80%. Notably, primer melting temperature (Tm) was not prioritized per RAA design principles. It is crucial to avoid the formation of secondary structures such as hairpins or dimers in primers and to ensure that there are no complementary sequences between upstream and downstream primers. The sequence of the *P. aeruginosa*-specific lasB gene (GenBank: JN118955.1) was retrieved from the NCBI database. Per the instructions provided in the RAA basic kit, four pairs of primers were formulated using the NCBI platform, and the optimal primer pairs were subsequently identified through experimental screening.

The crRNA, an RNA molecule carrying a specific sequence complementary to the target DNA, is at the heart of the CRISPR/Cas12a system. Like a key matching a lock, the crRNA recognizes and binds to the target DNA with high specificity. The design of the crRNA significantly influences the cleavage efficiency of Cas12a. According to the optimal primer amplification product fragments, we designed two crRNA sequences with PAM restriction in the length of 20–24 nt. The optimal primers and crRNAs are shown in Figures 2A,C. Detailed information on the primers, crRNAs, and ssDNA reporter gene sequences involved in the present study is shown in Supplementary Table S2.

### 2.6 RAA reaction system

The optimal primers were selected according to the recommended reaction system of the RAA kit (total volume of 50 μL). The system was as follows: One tube of dry powder, A buffer (25 μL), 2 μL of each upstream and downstream primer (10 μM), 13.5 μL of enzyme-free water, 5 μL of DNA template, and 2.5 μL of B buffer, for a total volume of 50 μL. To save the cost, the 50 μL total volume of the RAA was divided into five equal parts, so that the volume of each part was 10 μL (A buffer of 5 μL, 0.4 μL each of the upstream and downstream primers, 0.4 μL), and the volume of each part was 10 μL (A buffer 5 μL, 0.4 μL each of primers, 0.4 μL each of the primers). Buffer 5 μL, upstream and downstream primers 0.4 μL (10 μM) each, enzyme-free water 2.7 μL, DNA template 1 μL, B buffer 0.5 μL). The reaction was carried out at 37 °C for 15 min, and the amplification products were analyzed by 2% agarose gel electrophoresis.

### 2.7 CRISPR/Cas12a reaction system

In a 20 μL CRISPR/Cas12a reaction mixture, the following components were combined: 2 μL of NEBuffer r2.1 (10×), 2 μL of RAA expanded product, 500 nM of a fluorescent reporter molecule (single-stranded DNA, 5′-FAM-TTATT-BHQ1-3′), 50 nM LbCas12a (1 μM stock solution), and 50 nM crRNA. The total volume was adjusted to 20 μL with enzyme-free water. The reaction was monitored using a qPCR instrument, with the cutting process occurring at 37 °C for 30 min. During this period, fluorescence intensities were measured every 60 s. The fluorescence data obtained were subsequently analyzed using GraphPad Prism 10 (GraphPad Software, United States).

### 2.8 Constructing a rapid detection of *Pseudomonas aeruginosa* by glycerol one-pot RAA/CRISPR-Cas12a method

The glycerol one-pot RAA-CRISPR/Cas12a method fluorescence detection system has a total volume of 20 μL, which is divided into component A and component B. The system of component A included: NEbuffer r2.1 (10×), cas12a (200 nM), crRNA (200 nM), ssDNA (500 nM, 5′FAM-TTATT-BHQ1′3), 10% glycerol (This concentration was determined through optimization experiments shown in Figure 4A), and adjusted to a total volume of 10 μL with enzyme-free water. The system for component B consisted of 5 μL of A buffer, 0.4 μL of each of the upstream and downstream primers (200 nM), 2.7 μL of enzyme-free water, 1 μL of DNA template, and B buffer 0.5 μL. Component A was placed at the bottom of the EP tube, and then component B was added, 15 min of amplification at 37°C, followed by centrifugation with sufficient shaking and mixing. The cleavage process was monitored via a qPCR instrument, with the reaction proceeding at 37°C for a duration of 30 min, during which fluorescence intensities were measured at intervals of 60 s. In the one-pot reaction system, glycerol serves as a transient physical barrier, which ensures that the target DNA is preferentially amplified before the CRISPR/Cas12a cleavage reaction. The glycerol one-pot method has addressed the aerosol contamination issue caused by the traditional two-step method and also resolved the problem of reduced reaction efficiency associated with the conventional one-pot method. This approach has simplified the operational procedures.

The glycerol one-pot RAA-CRISPR/Cas12a method combines LFS, which had a total volume of 80 μL, and was divided into components A and B. The system of component A included: NEbuffer r2.1 (10×), cas12a (200 nM), crRNA (200 nM), ssDNA (500 nM, 5′FAM-TTATT-biotin'3), 10% glycerol, and supplemented with enzyme-free water to a total volume of 10 μL. The reaction system for component B comprises the following: 5 μL of Buffer A, 0.4 μL each of forward and reverse primers (200 nM), 2.7 μL of nuclease-free water, and 1 μL of DNA template. Component A was deposited at the bottom of a 1.5 mL microcentrifuge tube, followed by the addition of component B. The combined system was incubated at 37 °Cfor 15 min to complete Recombinase-aided Amplification (RAA). Following amplification, nuclease-free water was added to adjust the total reaction volume to 80 μL. The mixture was vortex-mixed for 10 s and centrifuged at 2000 *g* for 5 s, then subjected to a secondary incubation at 37 °C for 30 min (enabling Cas12a-mediated trans-cleavage). After mixing, insert the test strip and read the results within 10 min. The LFS assay is simple, rapid, and has the advantage of visual observation of the results.

### 2.9 Statistical analysis

Statistical analysis uses a one-sample t-test for comparison between two groups and one-way ANOVA for three or more groups. The results were expressed as mean ± standard deviation (SD). Each experiment should be repeated at least three times to ensure reliability. The negative control was the non-template control (NC). *P* < 0.05 was the statistical significance threshold, and all calculations were performed using GraphPad software.

## 3 Result

### 3.1 Construction of a *Pseudomonas aeruginosa* platform for rapid and reliable detection of glycerol one-pot RAA-CRISPR/Cas12a method

**FIGURE 1:**
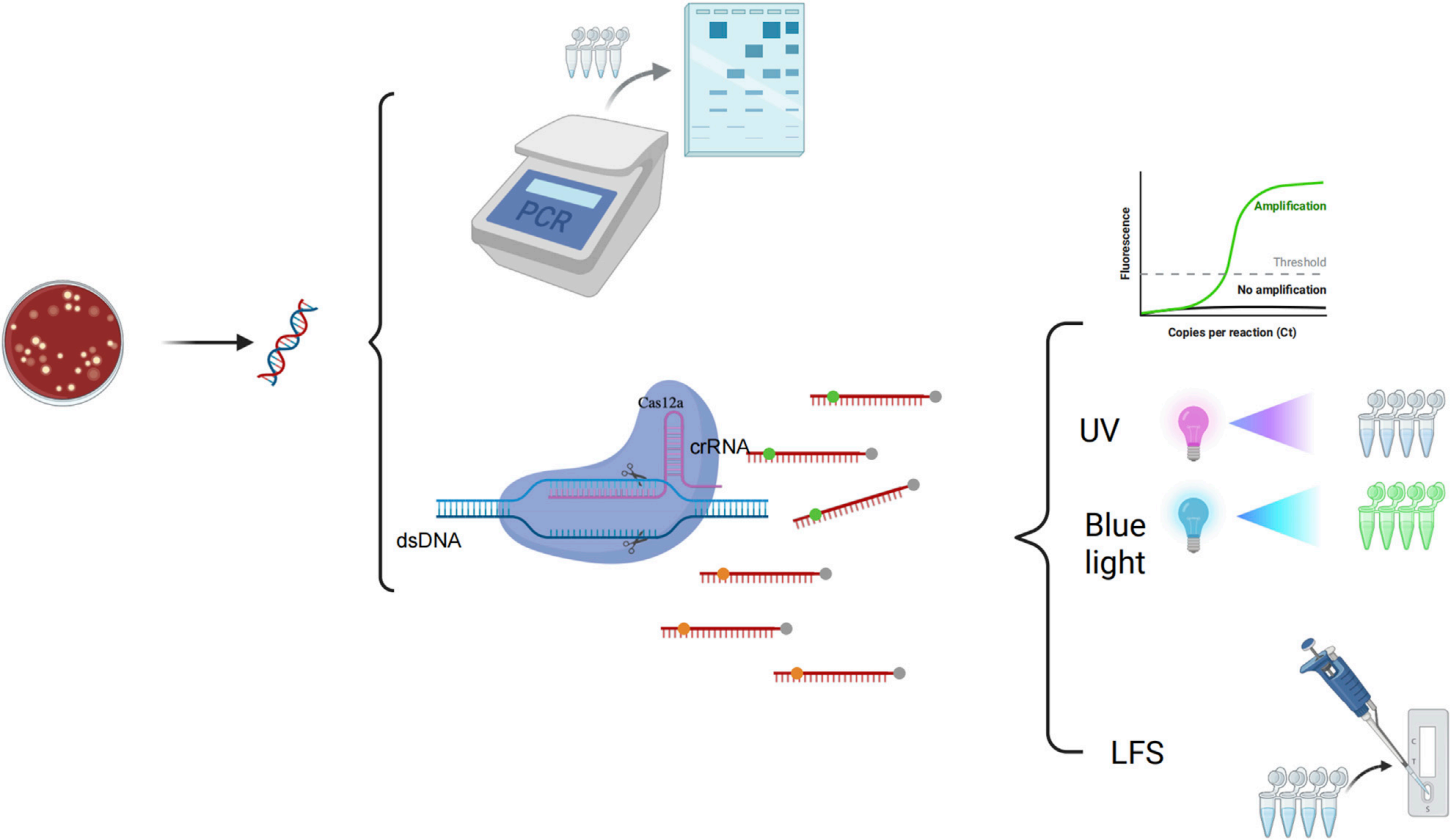
The principle of glycerol one-pot RAA/CRISPR-Cas12a method 1. DNA extraction of the PA. 2. Glycerol one-tube reaction: The system with a total volume of 20 μL is divided into two parts (10 μL of RAA system at the top of the tube and 10 μL of glycerol CRISPR system at the bottom of the tube). The reaction is carried out at 37°C for 15 min 3. The basic process of RAA amplification: The simplified principles are as follows: (1) Complex Formation: Recombinase binds to the primer to form a complex that recognizes homologous sequences in the target DNA. (2) Double-Strand Opening: The complex finds complementary sequences in the target DNA, causing the double-strand to separate. The primer then pairs with the target DNA. (3) Binding and Extension: Single-stranded binding protein stabilizes the open DNA, and a polymerase synthesizes a new complementary strand from the primer’s 3′end. (4) Cyclic Amplification: The new strand acts as a template for exponential amplification through repeated cycles. 4. The basic process of the CRISPR-Cas12a: The fluorescent probe (5′FAM-TTATT-BHQ1′3) was cleaved and the endpoint fluorescence value was recorded. The fluorescent probe (5′FAM-TTATT-biotin'3) was cleaved and the signal was detected using LFS. 6. PCR detection: The results were analyzed by 2% agarose gel electrophoresis. The glycerol one-pot RAA/CRISPR-Cas12a method yielded results that matched those of conventional PCR detection completely.

Currently, the conventional RPA-CRISPR/Cas12a detection system is divided into two steps: amplification and detection. However, aerosol contamination is easily generated when transferring amplification products. This may lead to false positive results. In this study, we used glycerol to physically separate the RAA system from the CRISPR system. This method solves the problem of aerosol pollution and realizes the one-pot detection of *P. aeruginosa*. The reaction process is shown in Figure 1. The assay approach is divided into three steps: the extraction of DNA, glycerol one-pot reaction, and visualization of the results. The extracted DNA serves as the template for Recombinase-aided Amplification (RAA). The amplification reaction is conducted at 37°C for 15 min. RAA is a thermostatic, rapid nucleic acid amplification technique that enables the rapid amplification of a large quantity of template DNA within a short period of time. The recombinase forms a stable complex with the primer. Subsequently, this recombinase-primer complex recognizes complementary sequences on the DNA template, thereby mediating duplex DNA unwinding. Concomitantly, single-stranded DNA-binding proteins (SSBs) coat the exposed strands to prevent reannealing and maintain structural stability. Following strand separation, the recombinase dissociates from the primer-DNA hybrid. DNA polymerase then catalyzes *de novo* strand synthesis through its binding to the 3′-hydroxyl terminus of the primer. After RAA amplification is complete, the mixture is mixed by shock centrifugation, and the CRISPR cutting reaction is carried out by incubating the mixture at 37°C for 30 min. The crRNA guides Cas12a to specifically bind to the target DNA, thereby forming a stable complex. Upon binding, Cas12a becomes activated and begins to nonspecifically cleave single-stranded DNA reporter molecules. Including a fluorescent stroke reporter probe (ssDNA-FQ, 5′FAM-TTATT-BHQ1′3), a FITC-biotin double-labeled probe (ssDNA-FB, 5′FAM-TTATT-biotin '3), respectively, for result visualization. Dual-mode readout platform: The reporter molecule ssDNA-FQ can be detected by the PCR instrument fluorescence equipment or observed under 365 nm ultraviolet (UV) or 475 nm blue light for color change. The reporter molecule, ssDNA-FB, is visually detected by the naked eye using the LFS. Drop the reaction mixture onto the sample pad of the test paper. As the liquid migrates, the gold nanoparticles coupled to the anti-FAM antibody bind to the FAM moiety. In the detection line, immobilized goat anti-mouse antibody captures anti-FAM antibodies that are conjugated with gold nanoparticles, resulting in the appearance of a red line (Yang Q. et al., 2024). In the control line, biotin is specifically bound by streptavidin and affinity, resulting in the formation of a red-colored band (Liao et al., 2024). A positive outcome is indicated by the presence of red bands in both the control line (C line) and the test line (T line) of the test strip, or by the absence of a red band in the control line (C line) while a red band appears in the test line (T line). Conversely, a negative result is characterized by a red band appearing in the control line (C line) and no visible coloration in the test line (T line) of the test strip.

The results showed that glycerol significantly improved the one-pot assay efficiency compared with the traditional one-step method, and the assay efficiency approximating the standard two-step method. The glycerol one-pot system solved the problem with nucleic acid cross-contamination due to frequent opening of the cap, and only when the target gene (lasB) was present, fluorescence was generated, and the test strip results were positive. Therefore, this demonstrated the feasibility of the glycerol one-pot method RAA-CRISPR/Cas12a system for PA detection in dual readout mode.

### 3.2 Improvement of glycerol one-pot RAA-CRISPR/Cas12a method

**FIGURE 2:**
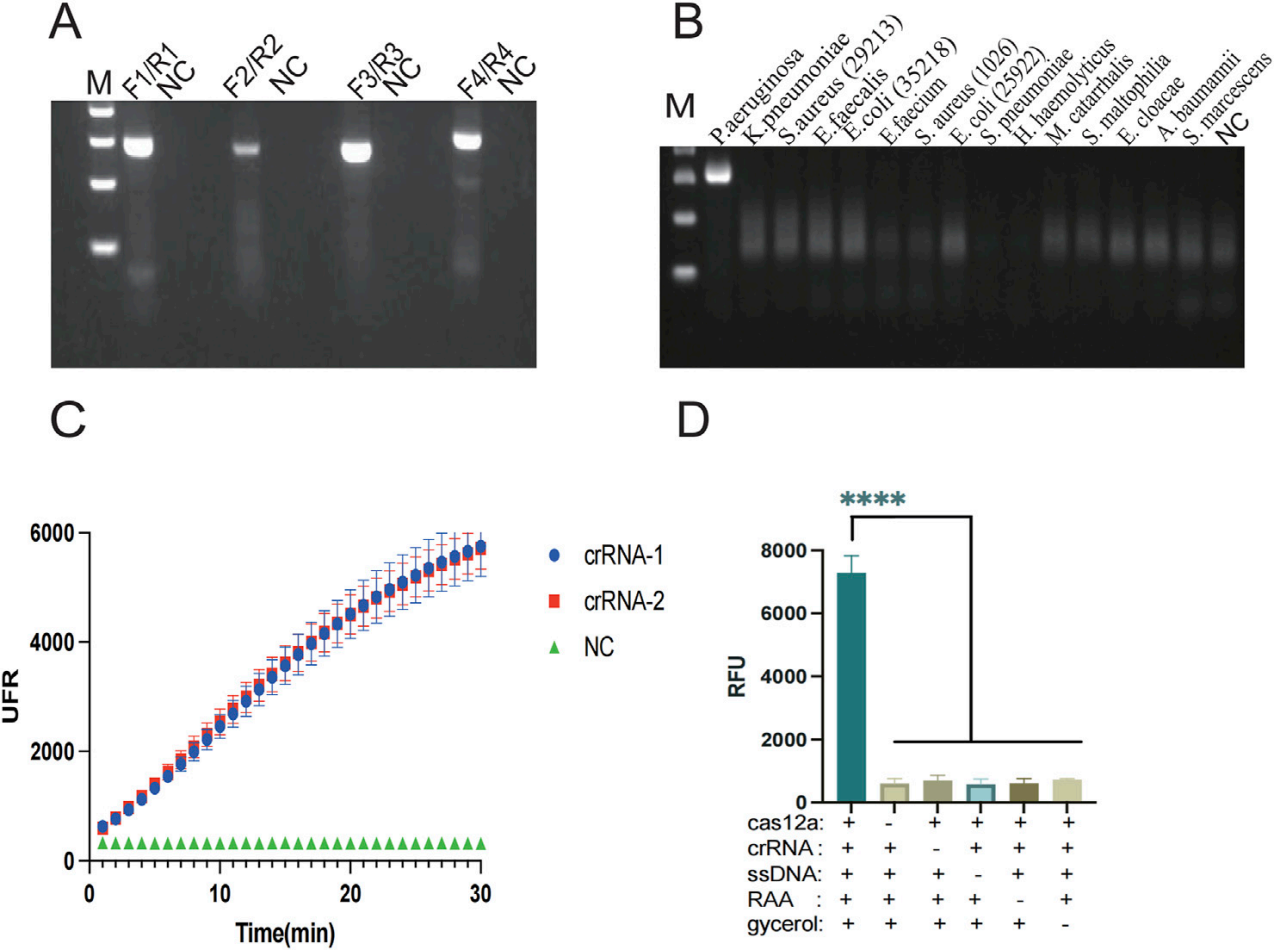
Screening of primers and crRNA by the glycerol one-pot method and experiments on the deletion of reaction components. **(A)** The best RAA primers screening: F4/R4 primers are the best RAA primers. **(B)** Specificity verification of F4/R4 primers. **(C)** The best crRNA screening: crRNA-1 is the best crRNA. **(D)** Experiment on missing reaction components, and record the endpoint fluorescence value. If there were no components such as cas12a, crRNA, ssDNA, RAA, and glycine, the result would not show obvious fluorescence. ****P < 0.0001 (n = 3). M, Marker. NC, negative control.

**FIGURE 3:**
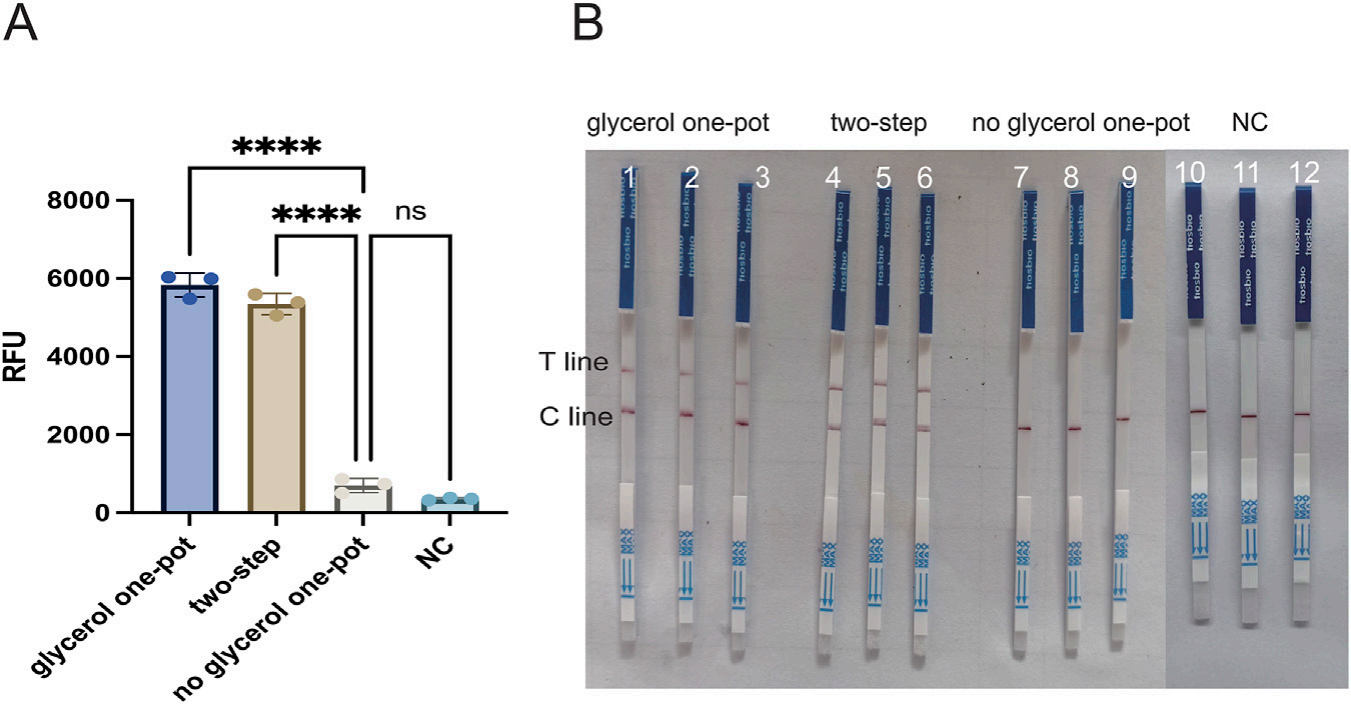
Experiment on missing reaction components of the glycerol one-pot method. **(A)** Feasibility verification of fluorescence detection: comparison among the glycerol one-pot method, the two-step method, and the no glycerol one-pot method. ****P < 0.0001 (n = 3). **(B)** Feasibility verification of LFS: comparison among glycerol one-pot method (1/2/3, 3 repetition), two-step method (4/5/6, 3 repetition), and traditional one-step method (7/8/9, 3 repetition), T line, test line. Cline, control line. NC, negative control (10/11/12, 3 repetition) ****P < 0.0001 (n = 3).

In the glycerol, one-pot RAA-CRISPR/Cas12a method detection, primers, and crRNA play a crucial role. Appropriate RAA primers are capable of effectively amplifying the template, and crRNA can complementarily pair with the target base and thus guide the Cas12a protein to recognize the target. Glycerol increases the viscosity of the liquid in the assay system and acts as a physical barrier to isolate the RAA system from the CRISPR cutting system. These three elements considerably affect its sensitivity and specificity. In order to achieve optimal amplification of RAA, four pairs of primers were designed with reference to the lasB gene, and the amplification products were analyzed by 2% agarose gel electrophoresis at the end of the RAA reaction. Primer 4 (F4/R4) showed the brightest band, while the target band was not observed for the control NC (Figure 2AB). To enable the crRNA to complementarily pair with the target base, two crRNAs (crRNA1, crRNA2) were designed in this paper. The efficiency of was crRNAs assessed via the CRISPR/Cas12a reaction, with the terminal fluorescence being captured for subsequent analysis. The findings revealed that the fluorescence values of crRNA-1 and crRNA-2 groups were markedly higher than those in the control group (*P* < 0.001) (Figure 2C). Therefore, primer 4 (F4/R4) and crRNA-1 were selected for subsequent detection. In this paper, the glycerol one-pot RAA-CRISPR/Cas12a method was validated for missing reaction components (Figure 2D). A clear color change and strong fluorescence can be observed only in reaction 1, which contains Cas12a protein, crRNA, ssDNA, target DNA, and glycerol. In the absence of any of these reaction components, the resulted is a failure to detect. To confirm the viability of the method, a glycerol single-tube detection method was established for comparison with two-step and conventional one-step methods. The glycerol one-pot method showed significantly higher fluorescence values compared to the traditional monotube method (*P* < 0.001). The incorporation of glycerol notably enhanced the detection capability in the monotube assay, bringing it nearly on par with the efficiency observed in the conventional two-step assay (Figure 3A). Based on the above validation, the glycerol one-pot method RAA-CRISPR/Cas12a detection system was successfully established. When combined with LFS, the detection lines of the glycerol single-step assay and two-phase assay were positive compared with the negative control, while the detection line of the traditional one-step assay was negative (Figure 3B).

**FIGURE 4:**
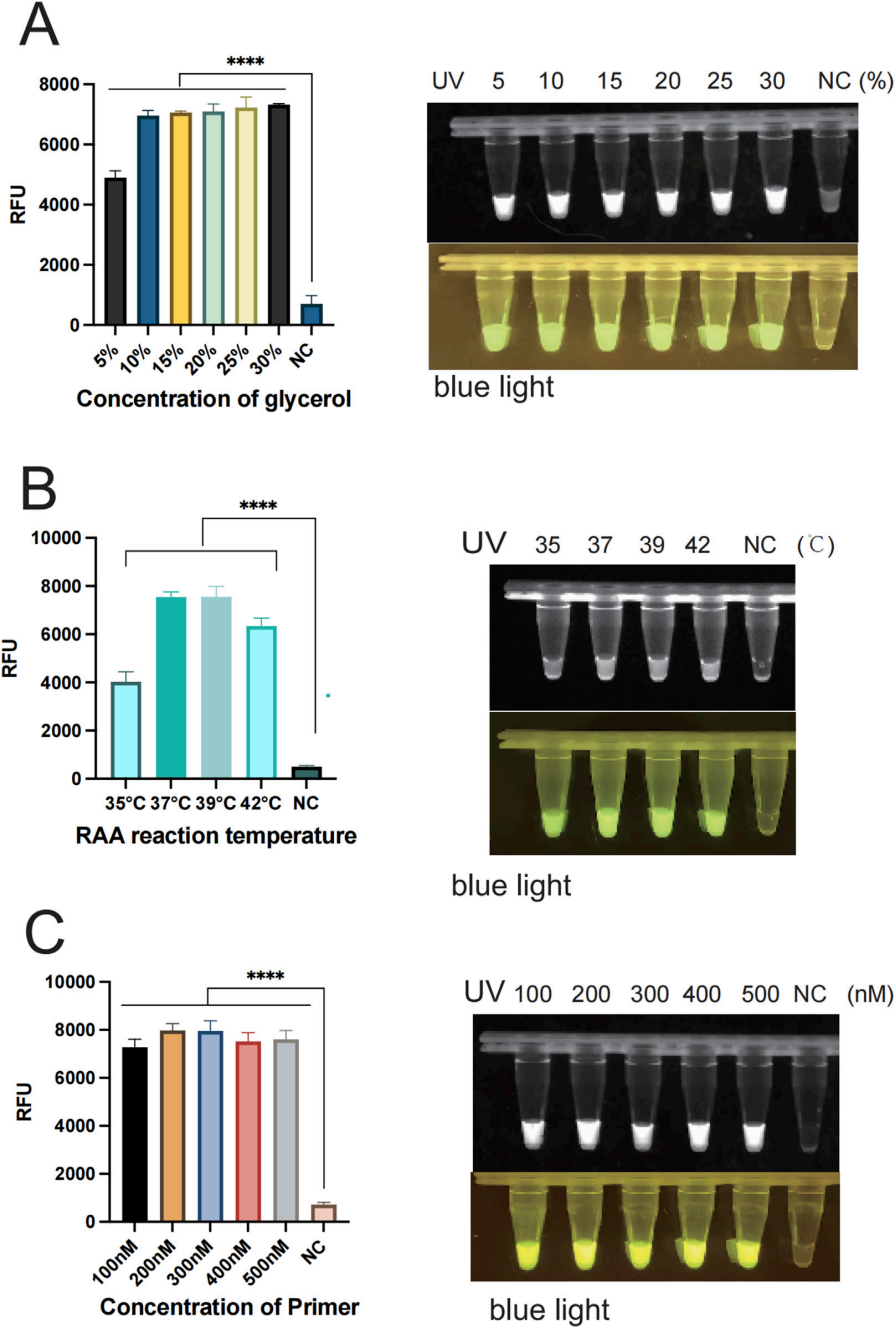
Optimization of each reaction component of the glycerol one-pot method. **(A)** The optimization of glycerol concentration with the optimal concentration being 10%. **(B)** Optimization of the reaction temperature for RAA, with the optimal temperature being 37°C. **(C)** Optimization of primer concentration, with the optimal primer concentration being 200 nM ****P < 0.0001 (n = 3). The above experimental results were all displayed under 365 nm ultraviolet (UV) and blue light. NC, negative control. Note: All subsequent experiments used 10% glycerol based on these optimization results.

**FIGURE 5:**
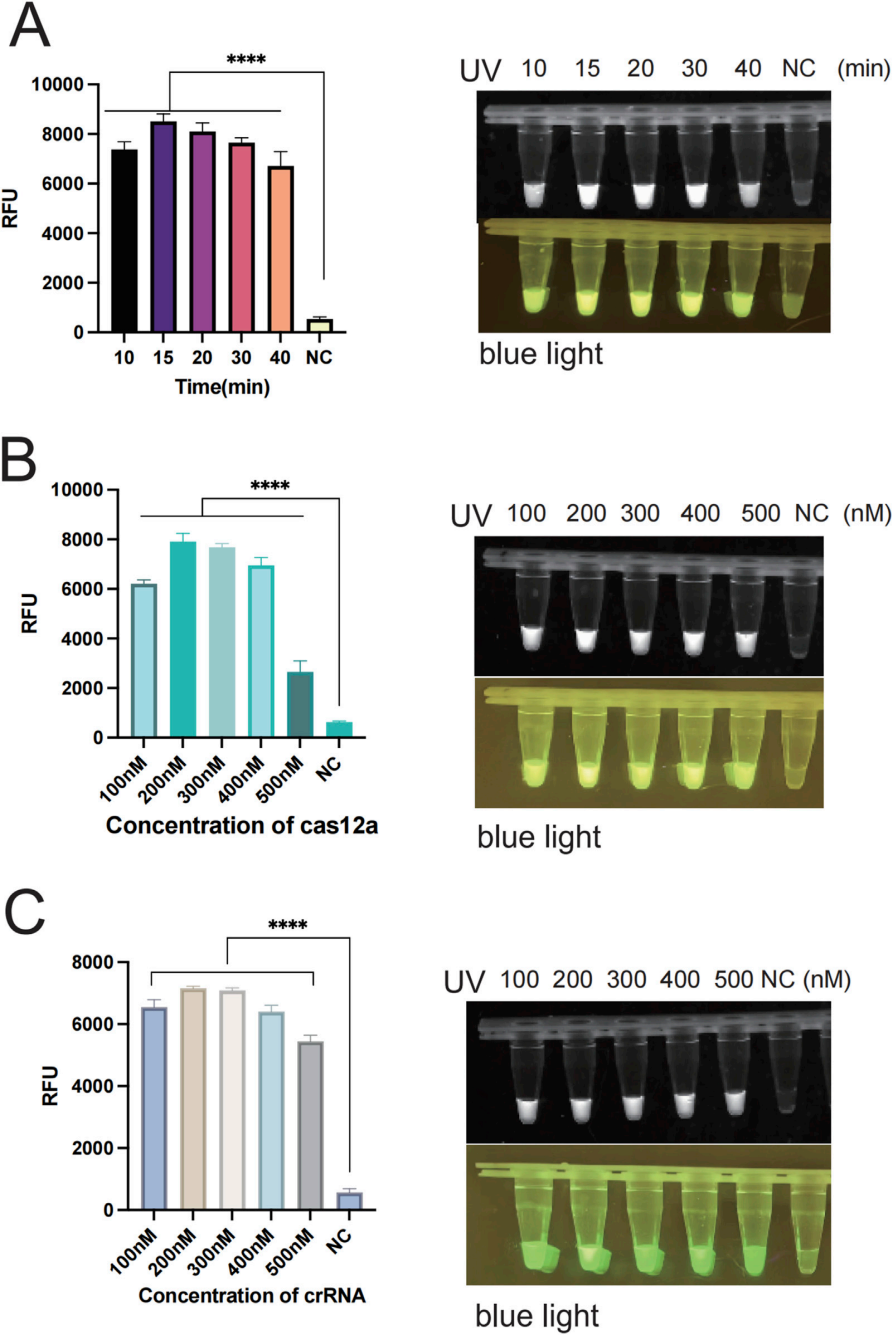
Optimization of each reaction component of the glycerol one-pot method. **(A)** RAA reaction time verification: The optimal reaction time is 15 min. **(B)** The optimization of cas12a concentration, with the optimal concentration being 200 nM. **(C)** The optimization of crRNA concentration, with the optimal concentration being 200 nM ****P < 0.0001 (n = 3). The above experimental results were all displayed under 365 nm ultraviolet (UV) and blue light. NC, negative control.

In order to enhance the accuracy of the outcomes and detection efficiency, this study was established to optimize the glycerol concentration, the RAA reaction system (temperature, time, and primer concentration), and the CRISPR/Cas12a system (Cas12a concentration, crRNA concentration, and ssDNA). To determine the optimal glycerol concentration in the glycerol one-pot method, different glycerol concentrations of 5%, 10%, 15%, 20%, 25%, and 30% were tested in this study. The fluorescence values were obviously enhanced at 5% (*P* < 0.001) and 10% (*P* < 0.001) compared with the control, as indicated by the results. The fluorescence values peaked at 10%, and although an increasing trend was observed at 15%, 20%, 25%, and 30%, no significant difference was observed compared to 10%. No visual differences were observed between 10% and 15%, 20%, 25%, and 30% under UV and blue light. Therefore, the optimal glycerol concentration was 10% (Figure 4A). To determine the optimal amplification reaction temperature for RAA, different temperatures were tested in this study (35°C, 37°C, 39°C, 42°C). The consequence showed that the fluorescence values were significantly higher (*P* < 0.001) at 35°C, 37°C, 39°C, and 42 °C as compared with negative control. The fluorescence curve peaked at 37 °Cand decreased at 42 °C. Therefore, the optimal temperature for RAA reaction was 37°C (Figure 4B). Primers play a primary role in the amplification of RAA, and in this study, different primer concentrations (100 nM, 200 nM, 300 nM, 400 nM, 500 nM) were tested. The results showed that the fluorescence values were significantly higher (*P* < 0.001) at 100 nM as compared to the negative control. Fluorescence values peaked at 200 nM, after which no significant difference was observed at 300 nM, 400 nM, or 500 nM. Therefore, the optimal primer concentration is 200 nm (Figure 4C). To determine the optimal amplification time for RAA, different time intervals of (10 min, 15 min, 20 min, 30 min, 40 min) were tested in our study. The findings indicated that the fluorescence values were significantly higher at 10 min compared with the negative control (*P* < 0.001). The fluorescence values peaked at 15 min and gradually decreased thereafter. However, there was no observable difference at 20 min or 30 min. Therefore, the optimal reaction time was 15 min (Figure 5A). Different Cas12a protein concentrations of (100 nM, 200 nM, 300 nM, 400 nM, 500 nM) were tested in this study. The outcome showed that the fluorescence value was observably higher at 100 nM compared with the negative control (*P* < 0.001). The fluorescence value peaked at 200 nM and then gradually decreased. Therefore, the optimal Cas12a protein concentration was 200 nm (Figure 5B). Similarly, we tested different concentrations of crRNA at (100 nM, 200 nM, 300 nM, 400 nM, 500 nM). The fluorescence value peaked at 200 nM and gradually decreased thereafter. Therefore, the optimal crRNA concentration is 200 nM (Figure 5C). Through the ssDNA concentration gradient optimization experiment, it was found that 500 nM is the optimal concentration for fluorescence detection, at which the fluorescence signal reaches a plateau (Supplementary Figure S1).

### 3.3 The glycerol one-pot method integrates the RAA-CRISPR/Cas12a method for *Pseudomonas aeruginosa* specificity validation

**FIGURE 6:**
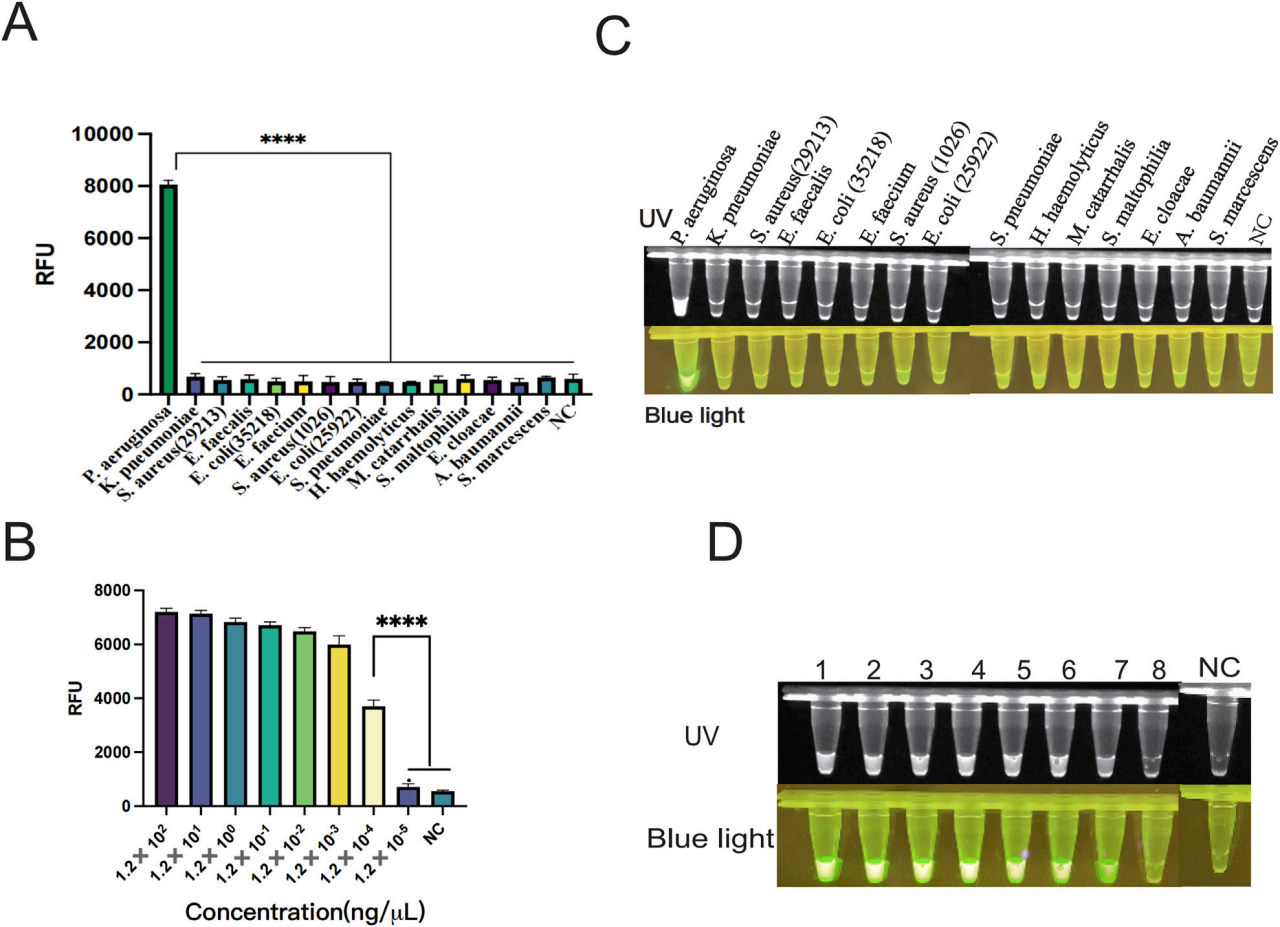
Verification of specificity and sensitivity of the one-pot glycerol method. **(A)** Specificity verification of PA by the one-pot glycerol method. Non-specific interference was carried out using *K. pneumonia, S. aureus* (ATCC 29213)*, E. faecalis, E. coli* (ATCC 35218)*, E. faecium, S. aureus* (ATCCBAA 1026)*, E. coli* (ATCC 25922)*, H. haemolyticus, S. pneumoniae, M. catarrhalis, S. maltophilia, E. cloacae,* **(A)** *baumannii, S. marcescens.* **(B)** Verification of the sensitivity of the one-pot glycerol method for detecting PA: The minimum detection limit of fluorescence detection is 1.20 × 10^−4^ ng/μL. **(C)** Fluorescence plots of the one-pot glycerol method for detecting PA specificity under ultraviolet and blue light. **(D)** Visualization results of the glycerol one-pot method sensitivity verification under ultraviolet and blue light. (UV and blue light,1-8 is PA concentration was serially diluted from 1.20 × 10^2^ ng/μL to 1.20 × 10^−5^ ng/μL), NC, negative control. ****P < 0.0001 (n = 3).

**FIGURE 7:**
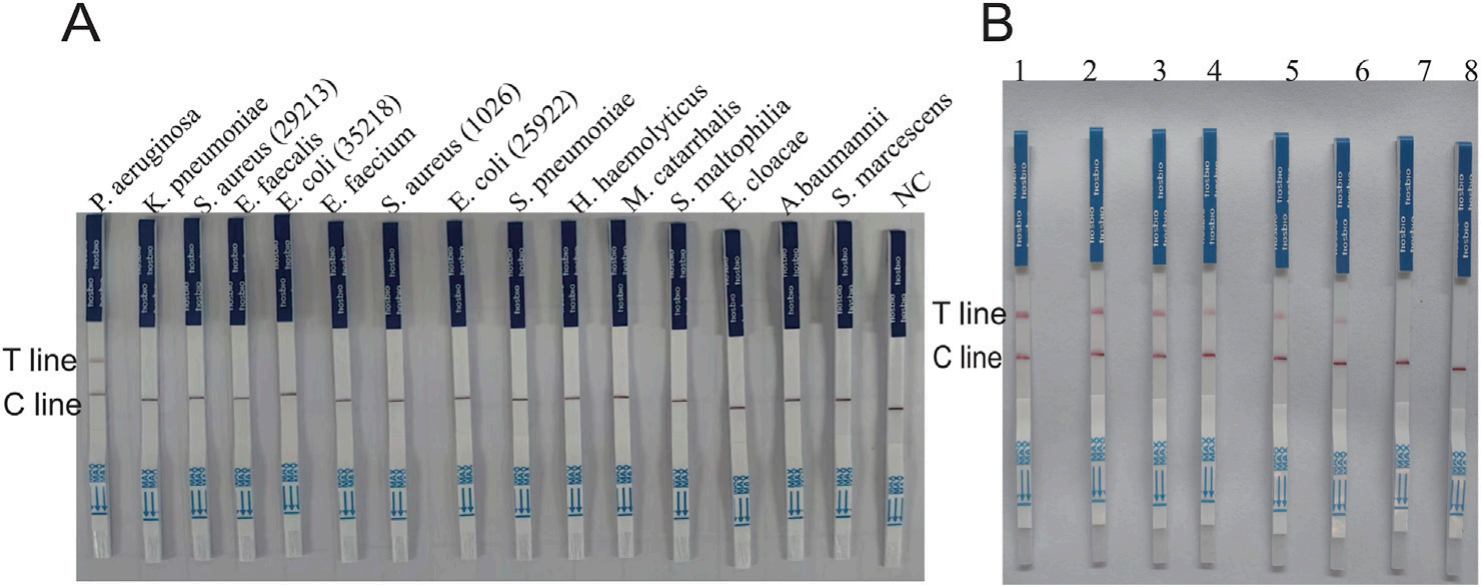
Verification of the specificity and sensitivity of the glycerol one-pot RAA-CRISPR/Cas12a -LFS. **(A)** Specificity verification of PA by the glycerol one-pot-LFS method. Non-specific interference was carried out using *K. pneumonia, S. aureus* (ATCC29213)*, E. faecalis, E. coli* (ATCC35218)*, E. faecium, S. aureus* (ATCCBAA 1026), *E. coli* (ATCC 25922), *H. haemolyticus*, *S. pneumoniae, M. catarrhalis, S. maltophilia, E. cloacae,* **(A)** *baumannii, S. marcescens.* **(B)** The sensitivity results of PA detected by the glycerol one-pot LFS method: the minimum detection limit of fluorescence detection is 1.20 × 10^−3^ ng/μL (1-7 is PA concentration was serially diluted from 1.20 × 10^2^ ng/μL to 1.20 × 10^−4^ ng/μL,8 = NC). T line, test line. Cline, control line. NC, negative control.

The genomic DNA of different strains was utilized to verify whether the established system has specificity. In this study, the specificity of the way was validated using standard strains including *Pseudomonas aeruginosa*, *Klebsiella pneumoniae*, *Staphylococcus aureus* (ATCCBAA 1026), *Enterococcus faecalis, Escherichia coli* (ATCC 35218), *Enterococcus faecium, Staphylococcus aureus* (ATCCBAA 1026), *Escherichia coli* (ATCC 25922), *Haemophilus haemolyticus*, *Streptococcus pneumoniae, Moraxella catarrhalis, Stenotrophomonas maltophilia, Enterobacter cloacae, Acinetobacter baumannii, and Serratia marcescens.* The results showed that only *P. aeruginosa* was able to produce significant fluorescent signals (Figure 6A). In addition, only the *P. aeruginosa* group produced fluorescence visible to the naked eye under UV light and blue light (Figure 6C). When united with the LFS assay, nothing but the *P. aeruginosa* group tested positive and showed consistency with this fluorescence assay (Figure 7A). The double cross-validation of the fluorescence assay and the LFS assay improved the accuracy of the assay. The experimental findings indicated that the glycerol one-pot method has high specificity for *P. aeruginosa* detection.

### 3.4 Glycerol one-pot method RAA-CRISPR/Cas12a method sensitivity analysis for *Pseudomonas aeruginosa*

The sensitivity of the glycerol one-pot RAA-CRISPR/Cas12a method for the recognition of *P. aeruginosa* was evaluated by constructing a standard plasmid (RTP6648-1–5). The concentration was serially diluted from 1.20 × 10^2^ ng/μL to 1.20 × 10^−5^ ng/μL; three replicate assays were performed for each dilution concentration. The endpoint fluorescence values were recorded after 30 min of CRISPR reaction, which revealed a gradual decrease in fluorescence values with decreasing concentration level of plasmid (Figure 6B). The fluorescence value was significantly higher in the 1.20 × 10^−4^ ng/μL group compared with the negative control (*P* < 0.001). Whereas, no observable difference was observed in fluorescence values in the 1.20 × 10^−5^ ng/μL group compared to the negative control (*P* > 0.05). The results indicated that our assay had a minimum detection line of 1.20 × 10^−4^ ng/μL for *P. aeruginosa*, the result matched the terminus fluorescence images seen under UV and blue light (Figure 6D). We also combined the LFS detection technique to further investigate the limit of detection (LOD) at different plasmid concentrations from 1.20 × 10^2^ ng/μL to 1.20 × 10^−4^ ng/μL (Figure 7B). The experimental results showed that the visual LFS detection technique of the glycerol one-pot RAA-CRISPR/Cas12a method could be sensitive enough to detect as low as 1.20 × 10^−3^ ng/μL. In conclusion, these consequences demonstrate the exceptional sensitivity of our detection method.

### 3.5 Assessment of the glycerol one-pot - RAA-CRISPR/Cas12a method for clinical sample detection

**FIGURE 8:**
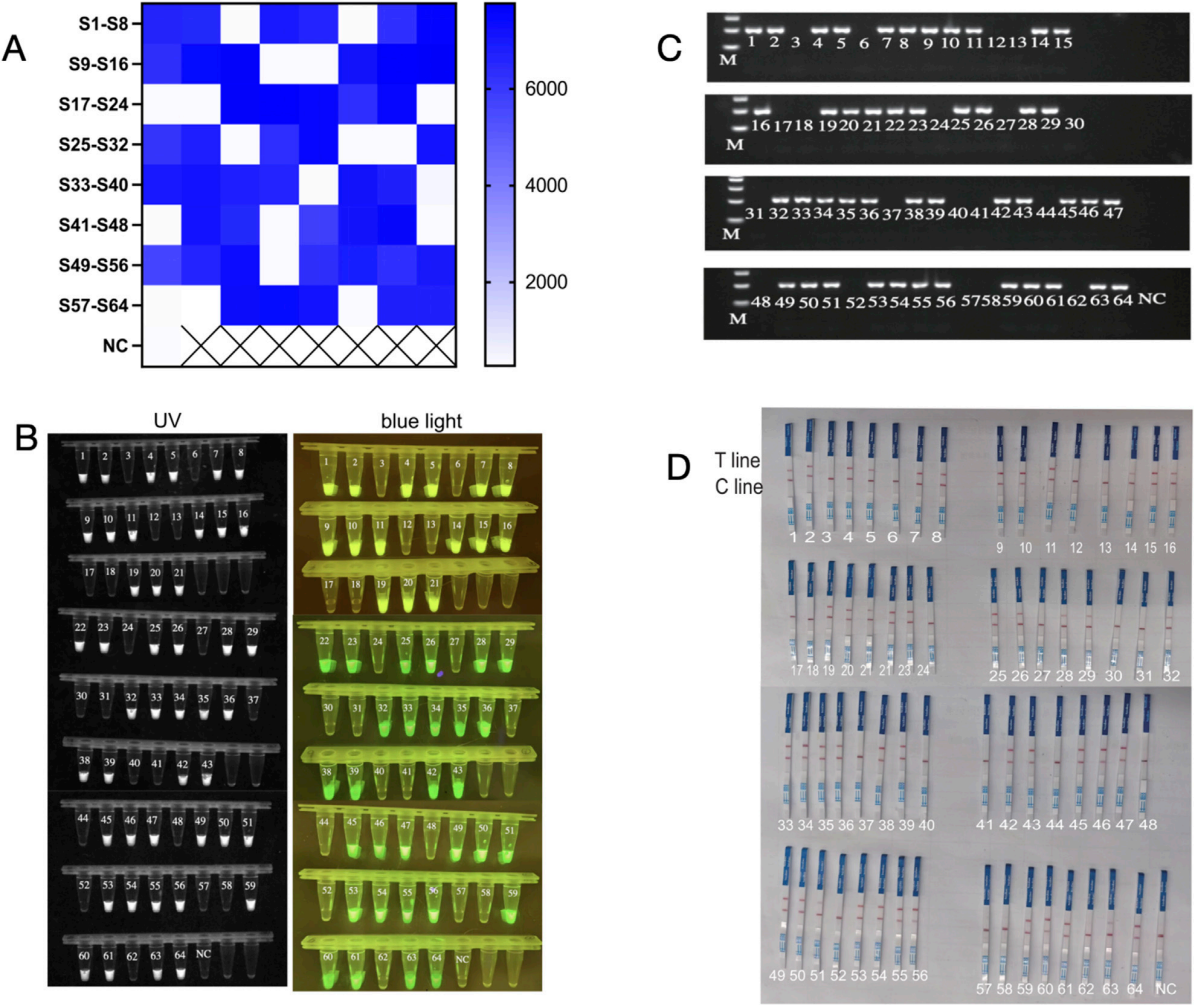
The glycerol one-pot - RAA-CRISPR/Cas12a method for clinical sample detection. A total of 64 clinical samples, including 45 cases of *P. aeruginosa*, and 19 cases of not *P. aeruginosa*. The glycerol one-pot method -RAA-CRISPR/Cas12a detection was adopted. **(A)** Heat map analysis of clinical sample detection. The endpoint fluorescence values were recorded and plotted as a heat map for analysis. S, sample. NC, negative control. ****P < 0.0001 (n = 3). **(B)** Visualization of clinical sample test results under ultraviolet and blue light. The results of the glycerol one-pot method were detected and visualized under ultraviolet and blue light. NC, negative control. ****P < 0.0001 (n = 3). **(C)** Clinical samples were detected by PCR. Clinical samples can be tested by the PCR method, and the results were analyzed by 2% agarose gel electrophoresis. NC, negative. M, Marker. **(D)** Assessment of the glycerol one-pot RAA-CRISPR/Cas12a -LFS method for clinical sample detection. Clinical samples can be detected by the LFS method, and the results can be observed by the naked eye. Cline, control line. T line, test line. NC, negative control.

In this study, the efficacy of the glycerol one-pot RAA-CRISPR/Cas12a method was evaluated using 64 clinical samples, and the outcomes were contrasted with PCR results. The clinical samples (n = 64) consisted of 45 *Pseudomonas aeruginosa*-positive specimens (positive specimen types included: sputum, alveolar lavage fluid, urine, prostate fluid, and ear canal secretions) and 19 *non-Pseudomonas aeruginosa* specimens. Heat map analysis indicated that 45 samples generated fluorescence, but 19 samples lacked significant fluorescence (Figure 8A), and the End-point fluorescent images under ultraviolet light and blue light (Figure 8B) were consistent with the results of agarose gel electrophoresis by PCR (Figure 8C).In addition, the glycerol one-pot RAA-CRISPR/Cas12a-LFS visualization assay detected 45 positive samples and 19 negative samples. These results were in agreement with the fluorescence data (Figure 8D). In the established glycerol single-tube RAA-CRISPR/Cas12a system, the detection rate of *P. aeruginosa* clinical samples was 100%.

## 4 Discussion

*P. aeruginosa* is a common opportunistic pathogen and is considered one of the causative agents of medically acquired infections. *P. aeruginosa* can cause respiratory, cutaneous, neurological, and bloodstream infections, and especially in immunocompromised individuals, may cause diseases. For example, otitis media inflammation, pneumonia, meningitis, and septicemia, are a serious health hazard and lead to significant economic losses, especially in resource-poor areas (Xu et al., 2024). Routine methods for the detection of *P. aeruginosa* include the bacterial culture method, polymerase chain reaction, and mass spectrometry techniques. However, these methods have the disadvantages of expensive instruments, complicated and time-consuming procedures, cumbersome operation steps, and the need for specialized laboratory equipment and well-trained technicians (Yaeger et al., 2025), which poses a primary barrier to the real-time monitoring, prevention, and control of *P. aeruginosa* infections, especially in resource-poor areas. Consequently, there is an urgent requirement for a simple, quick, sensitive, and specific detection method.

The advantages of RPA isothermal amplification technology with rapid amplification and the CRISPR/Cas12a system with high specificity and sensitivity. For the detection of numerous pathogenic bacteria, the RPA-CRISPR/Cas12a assay has been extensively utilized (Yin et al., 2025). For example, a method combining RPA with LFIA was used to detect *S. aureus* and *Vibrio parahaemolyticus*, with a detection limit of 4.6 × 10^2^ CFU/mL (Zhang et al., 2025b). The one-pot method is employed for rapid and sensitive detection of liver flukes in fecal samples (). A DR-CAMCas assay, integrating dual RPA and crRNA array-mediated CRISPR/Cas12a, enables accurate detection of *Staphylococcus aureus* through multiple signal outputs (Zhou et al., 2025). A two-step method, in which RAA amplification of the target is performed first, after which the product is introduced into the CRISPR system for reaction (Li et al., 2025). This two-step method is prone to aerosol contamination, leading to false positives (Zhong et al., 2025). In the traditional one-step method, due to the premature activation of the CRISPR system and cutting of the template during the initial phase of RAA amplification, the accumulation of target amplification is affected, which reduces the detection efficiency of the assay system (Zhang et al., 2024b; Gao et al., 2024). In order to solve the shortcomings of the two-step method and the traditional one-step method, we successfully constructed the glycerol one-pot method RAA-CRISPR/Cas12a assay. RAA and CRISPR are integrated into one system, and glycerol plays the role of isolation (Lin et al., 2022). This protocol synchronously solves the aerosol contamination problem caused by the two-step method and overcomes the low detection efficiency of the traditional one-step method, and the glycerol one-pot method achieves detection efficiency comparable to the standard two-step method. In order to maximize the detection efficiency, we optimized the glycerol concentration, RAA primer concentration, cas12a protein concentration, and crRNA concentration. We successfully devised a brief, fast, sensitive, and specific single-tube assay for the detection of *P. aeruginosa*. The method was optimized for 15 min of target amplification, reaction at 37 °C for 30 min, and the whole detection process took about 45 min. The assay achieves rapid target amplification and CRISPR detection within 45 min starting from purified DNA. Including nucleic acid extraction (approximately 30 min), the total sample-to-result time is approximately 75 min. Therefore, the glycerol one-pot RAA-CRISPR/Cas12a method has great potential for future field detection.

The glycerol one-pot RAA-CRISPR/Cas12a method in this study utilized a dual readout mode. First, fluorescence detection mode: RAA was amplified at 37°C for 15 min and then incubated at 37°C for 30 min for the CRISPR reaction, the fluorescence value was recorded every 60 s, and the detection limit was 1.20 × 10^−4^ ng/μL. Second, amplification was performed at 37°C for 15 min, replenished to 80 μL by adding enzyme-free water to carry out a sufficiently shocked mixing centrifugation and then followed by incubation at 37 °Cfor 30 min, mixing The test strip was inserted and the result was read within 10 min, with a detection limit of 1.20 × 10^−3^ ng/μL. The lateral flow strip assay system is a simple, rapid, and visually observable system, which is more suitable for immediate detection in resource-poor regions than the PCR assay (Wu et al., 2025). This dual-readout mode produces visible fluorescence and a positive test line of the test strip only With the target gene (lasB*)*is present, and this cross-validation mode reduces false positives of the assay. Therefore, the glycerol one-pot method assay possesses the benefits of simplicity, speed, sensitivity, and specificity. The light-controlled one-pot method requires the design and synthesis of NPOM-modified crRNA, and NPOM modification is more expensive (Hu et al., 2023). The one-pot microarray method (Ren et al., 2024) is also more complicated in its microarray design. The glycerol one-pot method, in contrast, merely necessitates adding inexpensive glycerol to the system, and its detection efficiency is nearly on par with the two-step method. Qiu et al. developed a *Pseudomonas aeruginosa*-based on CRISPR/Cas12b single-component treatment for one-step detection (Qiu et al., 2023). The single-tube LAMP-Cas12b method achieves detection in a single reaction tube, significantly simplifying the operational steps. However, this method requires a reaction temperature of 55 °C and multiple pairs of primers, which increases the complexity of the reaction. In contrast, the glycerol one-pot method described in this paper only needs a reaction temperature of 37 °C and can rapidly amplify nucleic acids with just one pair of primers. For a detailed comparison of the two methods, please refer to Supplementary Table S3.

Although our detection scheme realizes single-tube detection, simplifies the operation and reduces aerosol contamination, there are some limitations. For example, nucleic acid extraction is required before the experiment, which increases the complexity of the pre-treatment operation. Although nucleic acid extraction currently extends the total time, our combined 75 min workflow remains notably faster than standard clinical germiculture; for instance, RT-qPCR typically requires ≥120–180 min (including extraction). Our work focused on validating the core RAA-CRISPR detection speed and functionality starting from purified DNA. We recognize the critical need for direct sample testing without extraction, particularly in resource-limited settings, and will prioritize developing extraction-free workflows in follow-up studies. The concentration of glycerol needs to be precisely controlled, and too high or too low a concentration of glycerol will affect the detection efficiency. The spatial isolation effect of glycerol is the core of our design, but its potential auxiliary functions (such as maintaining enzyme activity and regulating local viscosity) will be the focus of our future exploration.

## 5 Conclusion

In conclusion, we establish the glycerol one-pot-RRA-CRISPR/Cas12a method for rapid detection of *P. aeruginosa*. The method enables easy, rapid, sensitive, and specific detection of *P. aeruginosa* at a common temperature. The method achieves a detection limit of 1.20 × 10^−4^ ng/μL in 30 min at 37°C by fluorescence detection and 1.20 × 10^−3^ ng/μL in 30 min at 37°C by LFS-based detection. Results can be read by collecting fluorescent signals under UV or blue light, or by using the LFS technique. In addition, the method is easy to operate, eliminates the need to open containers to transfer liquids, effectively prevents aerosol contamination, and eliminates the need for elaborate temperature control systems. It can achieve rapid diagnosis of *Pseudomonas aeruginosa*.

## Data Availability

The raw data supporting the conclusions of this article will be made available by the authors, without undue reservation.
