# Supplementary material for: Rapid detection of Pseudomonas aeruginosa by glycerol one-pot RAA/CRISPR-Cas12a method
Source: Front Chem. 2025 Jul 25;13:1654270. doi: 10.3389/fchem.2025.1654270 (PMC12331700; doi:10.3389/fchem.2025.1654270)
Supplement: Supplementary file 1 [file DataSheet1.pdf]

Supplementary materials:

Table S1. Information on pathogenic microorganisms used in this study

Name	Category number	Source
P.aeruginosa	ATCC27853	American Type Cultur Collection
K.pneumoniae	ATCC700603	American Type Cultur Collection
S.aureus	ATCC29213	American Type Cultur Collection
E.faecalis	ATCC29212	American Type Cultur Collection
E.coli	ATCC35218	American Type Cultur Collection
E.faecium	GDMCC1.388	Guangdong Microbial Culture Collection Center
S.aureus	ATCCBAA1026	American Type Cultur Collection
E.coli	ATCC25922	American Type Cultur Collection
S. pneumoniae	ATCC49619	American Type Cultur Collection
H. haemolyticus	ATCC33390	American Type Cultur Collection
M. catarrhalis	-	Clinical isolates
S. maltophilia	-	Clinical isolates
E. cloacae	-	Clinical isolates
A. baumannii	-	Clinical isolates
S. marcescens	-	Clinical isolates

Table S2. Sequences of Primers, crRNA, and ssDNA reporters involved in this study

Primer	base sequence	base number
PA-primer1	F:CAACCAGAAGATCGGCAAGTACACCTACG	29
	R:TAGTGCACCTTCATGTACAGCTTGTGGGTC	30
PA-primer2	F:GCAACCAGAAGATCGGCAAGTACACCTACG	30
	R:GTAGTGCACCTTCATGTACAGCTTGTGGGT	30
PA-primer3	F:GAAGAAGGTTTCTACGCTTGACCTGTTGTT	30
	R:GCCGGGACCCTTGACTTCGGTGATGGCTT	29
PA-primer4	F:AAGCCATCACCGAAGTCAAGGGTCCCGGC	29
	R:TCCCACTGATCGAGCACTTCGCCGGTCTT	29
crRNA1	UAAUUUCUACUAAGUGUAGAUGUCGCCAAC AUCGCUGCCGA	46
crRNA2	UAAUUUCUACUAAGUGUAGAUUCAUUCUCG GUCUUGCGGCC	46
PCR-primer	F:TGTCCAAACTCCCCAGCAAG	20
	R:CCTTGACTTCGGTGATGGCT	20
SSDNA	5‘FAM-TTATT-BHQ1’3	6
	5‘FAM-TTATT-biotin’3	6

Table S3. Our one-pot glycerol method was compared with LAMP-Cas12b

**Parameter**	**Glycerol RAA/Cas12a**	LAMP-Cas12b (Qiu et al.)
Reaction temperature	37℃	55℃
Result reading method	4 modes (Fluorescence/UV/Blue light/LFS)	2 modes (Fluorescence reader and a lateral flow biosensor)
Reaction time	45 min	60 min
Primer	A pair of primers	Three pairs of primers
Detection limit (DNA)	1.20×10^{-4} ng/μL (fluorescence)	10 copies/mL
Easy operating	Single-tube detection reduces operation steps and contamination risks	Single-component treatment reduces operation steps and contamination risks

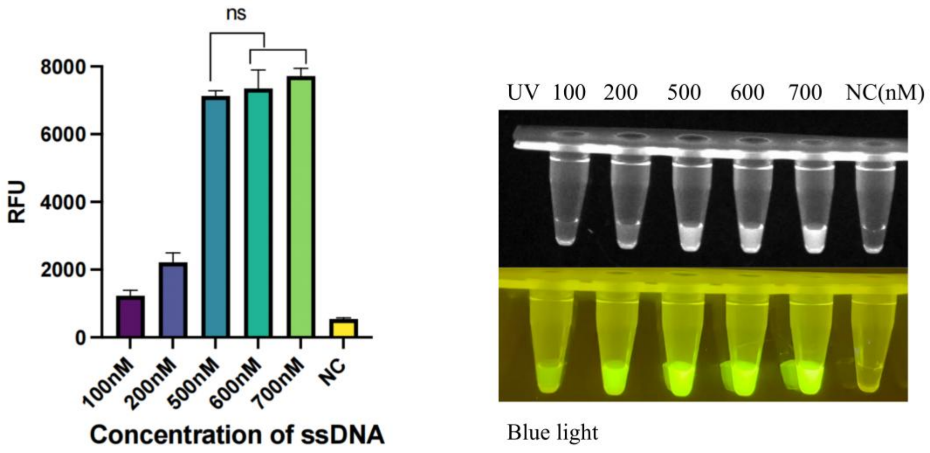

Figure S1. Optimization of ssDNA concentration. Through the ssDNA concentration gradient optimization experiment (100–700 nM), it was found that 500 nM is the optimal concentration for fluorescence detection, at which the fluorescence signal reaches a plateau. As can be observed from the data, when the ssDNA concentration reaches 500 nM, the fluorescence signal is significantly enhanced. Further increases to 600–700 nM only bring about marginal improvements. This indicates that 500 nM is sufficient to meet the system's requirements. Increasing the concentration beyond this point will not significantly enhance sensitivity and may instead increase the risk of non-specific background noise. UV, ultraviolet. NC, negative control.
